# Climate and Weather Impact Timing of Emergence of Bats

**DOI:** 10.1371/journal.pone.0042737

**Published:** 2012-08-02

**Authors:** Winifred F. Frick, Phillip M. Stepanian, Jeffrey F. Kelly, Kenneth W. Howard, Charles M. Kuster, Thomas H. Kunz, Phillip B. Chilson

**Affiliations:** 1 Ecology and Evolutionary Biology, University of California Santa Cruz, Santa Cruz, California, United States of America; 2 School of Meteorology and Atmospheric Radar Research Center, University of Oklahoma, Norman, Oklahoma, United States of America; 3 Oklahoma Biological Survey and Department of Biology, University of Oklahoma, Norman, Oklahoma, United States of America; 4 National Severe Storms Laboratory, National Oceanic and Atmospheric Administration, Norman, Oklahoma, United States of America; 5 Center for Ecology and Conservation Biology, Department of Biology, Boston University, Boston, Massachusetts, United States of America; University of Western Ontario, Canada

## Abstract

Interest in forecasting impacts of climate change have heightened attention in recent decades to how animals respond to variation in climate and weather patterns. One difficulty in determining animal response to climate variation is lack of long-term datasets that record animal behaviors over decadal scales. We used radar observations from the national NEXRAD network of Doppler weather radars to measure how group behavior in a colonially-roosting bat species responded to annual variation in climate and daily variation in weather over the past 11 years. Brazilian free-tailed bats (*Tadarida brasiliensis*) form dense aggregations in cave roosts in Texas. These bats emerge from caves daily to forage at high altitudes, which makes them detectable with Doppler weather radars. Timing of emergence in bats is often viewed as an adaptive trade-off between emerging early and risking predation or increased competition and emerging late which restricts foraging opportunities. We used timing of emergence from five maternity colonies of Brazilian free-tailed bats in south-central Texas during the peak lactation period (15 June–15 July) to determine whether emergence behavior was associated with summer drought conditions and daily temperatures. Bats emerged significantly earlier during years with extreme drought conditions than during moist years. Bats emerged later on days with high surface temperatures in both dry and moist years, but there was no relationship between surface temperatures and timing of emergence in summers with normal moisture levels. We conclude that emergence behavior is a flexible animal response to climate and weather conditions and may be a useful indicator for monitoring animal response to long-term shifts in climate.

## Introduction

Changes in climate can affect animal and plant populations in numerous ways [1]. Much recent attention has focused on how increased warming correlates to changes in phenology [2], [3] and its potential for de-coupling resource-consumer interactions [4], [5]. Seasonal changes in climate at local and regional scales can also have profound influences on demographic dynamics of populations for species with narrow thermodynamic tolerances or those existing at range edges [6], [7]. Both climate and weather likely have direct and indirect effects on animal populations [8] and understanding how animals respond to shifts in climatic conditions is important for determining long-term impacts of global climate change on ecosystems.

One limitation to understanding how climate affects animal behavior is lack of long-term datasets that adequately measure behavioral response at the time scales necessary to detect responses to shifts in climate. Use of remote sensing data to measure changes in primary productivity (e.g. Normalized Difference Vegetation Index) provide a means to assess changes in vegetation communities [9]. These data sources typically lack information on vertebrate or other consumer response, limiting ability to retrospectively analyze responses of animals to variation in climate.

The data archive maintained by the National Climatic Data Center (NCDC) of national networked weather radars (collectively known as NEXRAD) contains signals of animals aloft in the aerosphere going back to the early 1990s [10]. The NEXRAD radar archive is arguably one of the largest treasure troves of biological information that is relatively untapped by ecologists [11]. For populations that have large aggregations associated with known point localities on the ground (e.g. cave-roosting bats and colonially-roosting birds), the radar archive contains information on changes in daily behavioral patterns, such as when animals take flight, that can readily be used to assess changes in phenology [12] or response to daily or seasonal climate conditions.

Timing of emergence to forage by bats is an adaptive behavior that has important fitness consequences in terms of trade-offs between increased risk of predation or competition with diurnal aerial insectivores and forfeiting foraging opportunities during peak prey availability [13], [14], [15]. Bats that leave a roost early face greater risk of predation, but increase foraging time during crepuscular periods, when aerial insect availability may be high [14], [16]. Several studies have demonstrated that foraging habits and reproductive condition of bats influences onset of emergence in ways that support this hypothesis [13], [14], [17], [18]. In particular, lactating females are the most energetically stressed and therefore should emerge earlier if energetic demands outweigh costs of increased risk of predation. This pattern has been demonstrated in several species [14], [17]. The hypothesis that increased physiological stress results in earlier emergence times leads to predictions about how climate variation may influence emergence behavior of bats. Specifically, if climate or weather conditions cause physiological stress then bats may emerge earlier during periods associated with environmental stress, such as drought.

Here, we test whether emergence behavior of Brazilian free-tailed bats (*Tadarida brasiliensis*) during the maternity season is associated with variation in summer drought conditions over the past 11 years. Drought causes physiological stress for many bat species, particularly in summer months when bats are reproductively active [6], [19]. Drought is associated with lower prey availability [20] and water balance stress in bats [19]. We predicted that Brazilian free-tailed bats would emerge to forage earlier during droughts if physiological stress from extrinsic climatic conditions has a strong influence on emergence behavior. Timing of emergence may also vary with daily weather conditions, such as surface temperature. Nocturnal moth activity is generally positively correlated with temperature, such that hotter nights should correspond with higher prey availability [21]. We predict that bats would emerge later on days with higher surface temperatures because foraging success should be higher with increased temperature if prey are more plentiful when it is warm and bats can emerge later in the evening and still meet energetic needs. The relationship between daily temperature and onset of emergence may depend on summer climatic condition, such that in drought conditions the influence of daily temperature may be different than in normal or unusually moist years. By analyzing variation in emergence behavior at a seasonal and daily scale, we aim to determine the flexibility of response to variation in weather and climate that leads to insights about how long-term climate shifts could impact animal populations.

## Methods

To compare bat emergence behavior with daily and seasonal meteorological conditions, it was first necessary to establish a record of the time of emergence for a selection of bat colonies. Brazilian free-tailed bats disperse nightly in dense columns from cave and bridge roosts and forage at high altitudes (300–2500 m AGL) over large spatial extents that are regularly detected by the NEXRAD network of weather surveillance radars [22], [23]. Although the NEXRAD network is designed to detect precipitation and weather events, these weather radars have the capacity to monitor and survey aerial animals, including birds, bats, and arthropods [10], [11]. A long-running archive of NEXRAD data is available at NCDC (www.ncdc.noaa.gov), including all three conventional radar products: radar reflectivity factor (Z), radial velocity (v_r_), and spectrum width (σ_w_). The measure of backscattered intensity, radar reflectivity factor (Z), can be directly related to the number of aerial organisms occupying the aerosphere [24], and therefore is the appropriate measure for identifying colony emergence.

**Figure 1:**
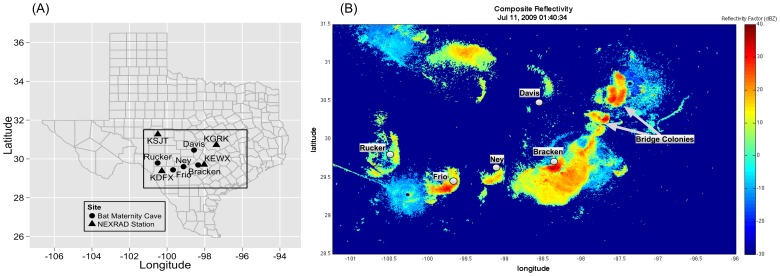
Study Region and Radar Imagery. (A) The study domain outlined in black in south-central Texas and showing locations of five maternity colonies of Brazilian free-tailed bats (*Tadarida brasiliensis*) and four NEXRAD radar installations that were used in mosaicking radar reflectivity data. (B) Sample image from mosaicked radar reflectivity across the spatial domain. False color scale in dBZ shows characteristic emergence ring signatures (warmer colors of yellow to red) at the five maternity sites for July 11^th^, 2009 at 01:40:34 UTC, corresponding to 20:40:34 local time on July 10^th^, 2009.

We chose five maternity colonies of Brazilian free-tailed bats in south-central Texas, which are regularly detected by radar (Fig. 1). Because the altitude of the radar sampling volume increases with range from the radar, maternity colony sites were restricted to be within 110 km of a NEXRAD station to ensure adequate height coverage of emergences [25], [26]. Bridge-dwelling colonies were not included to ensure consistency among samples and eliminate any influences introduced by anthropogenic roost structures [23].

NCDC stores NEXRAD radar products from individual radars in polar coordinates. To provide the best spatial coverage of the selected caves, we chose four of the surrounding NEXRAD installations (KSJT, KGRK, KEWX, and KDFX) for our analysis (Fig. 1). Using a radar-merging algorithm, we meshed radar reflectivity factor data from the four radars onto a common Cartesian grid [27], [28], [29], [30]. From this three-dimensional grid of radar reflectivity factor values (Z), we projected the maximum value in height to the surface, a method known as radar compositing [31]. The result is a two-dimensional map of maximum reflectivity values in the vertical column, known as composite reflectivity (CREF). The spatial resolution of our final CREF values was 500 meters by 500 meters, and the temporal update time was five minutes. CREF data at coarser resolution (1 km×1 km grid cells) covering the continental USA since 2008 at five-minute temporal resolution are available through the SOAR (Surveillance Of the Aerosphere using weather Radar, http://soar.ou.edu) web portal. Data generated for this analysis were processed by special request by the National Severe Storms Laboratory that hosts SOAR. We chose to focus on data from the period of June 15 through July 15, corresponding to peak lactation period for Brazilian free-tailed bats [17], from 2001 to 2011 to acquire a sufficiently long time series for our purposes. For meteorological applications, values of radar reflectivity factor are typically reported in logarithmic units, dBZ. To relate the reflectivity factor to bioscatter in the aerosphere, we converted to linear units of Z [24].

**Figure 2:**
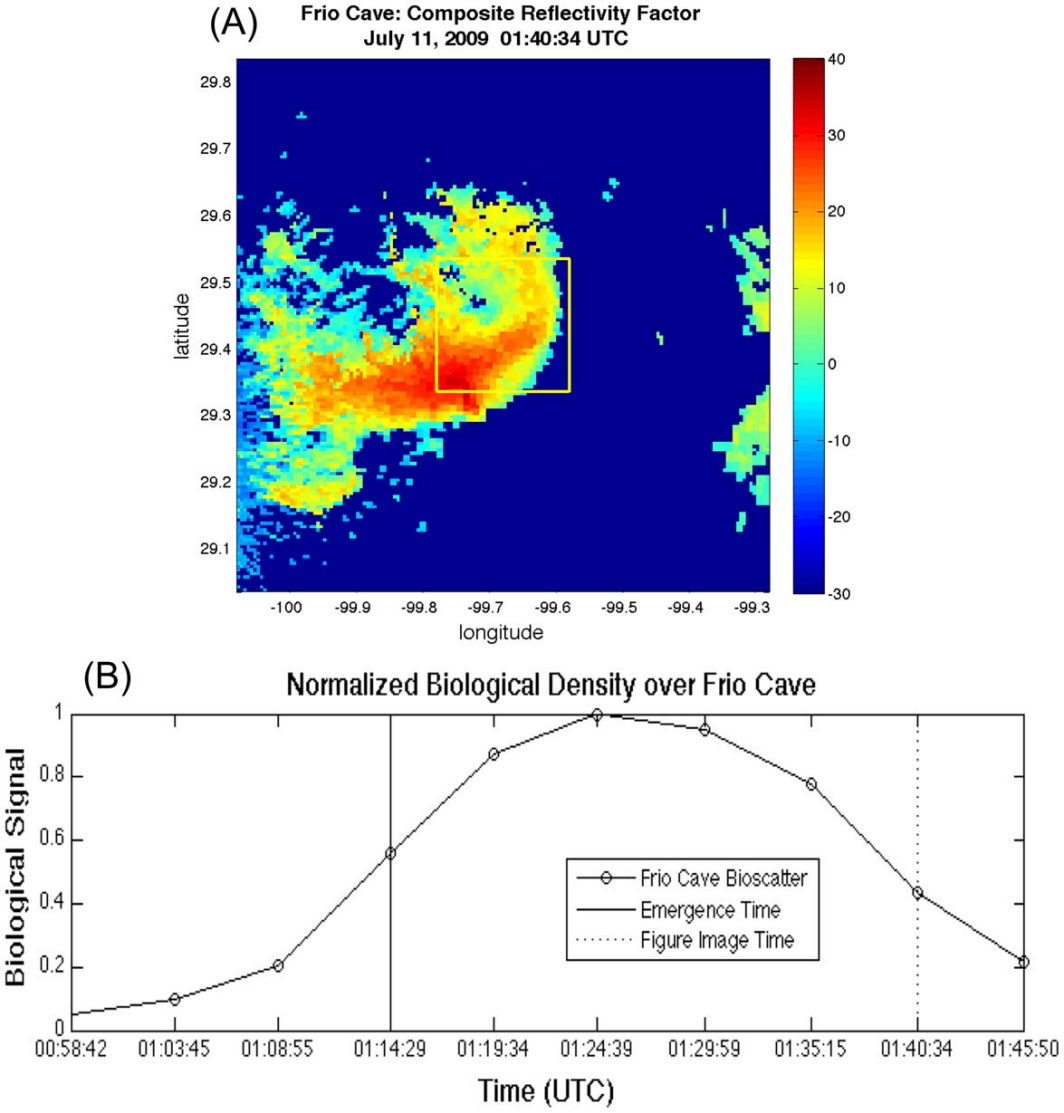
Radar-Based Methods for Determining Timing of Emergence. (A) Brazilian free-tailed bats (*Tadarida brasiliensis*) emerging from Frio Cave on July 11^th^, 2009 at 01:40:34 UTC shown in radar imagery in dBZ. The spatial bounding box (40×40 pixels) used to calculate the index of total airborne biological density over the cave is outlined in yellow. (B) A normalized time series of summed linear Z values was used to determine the steepest slope (maximum dZ/dt) to determine the peak time of emergence used in statistical analyses (solid vertical line). The vertical dotted line marks the time associated with image shown in Figs. 1 and 2a.

To determine emergence time for each colony on each day, we defined a 40 by 40 pixel (20 km by 20 km) spatial domain centered on each of the five cave locations (Fig. 2a). A broad spatial domain surrounding each cave was required because variability in flight direction during emergence sometimes results in bats literally flying “under the radar” causing horizontal displacement of where bats rise to detectable altitudes. The domain size was chosen, after visual inspection of radar imagery, to be large enough to allow for spatial variability in location where emergence was detected, while remaining small enough to avoid contamination from other nearby bat colonies. Each of the five cave domains consists of 1600 pixels (40×40). Our analyses are based on linear values of radar reflectivity factor for each pixel. At each time step, we summed the 1600 Z values to obtain a single measure of the total biological density in the aerosphere over each of the five caves. By repeating this process at each five-minute time step, we obtained a time series of the index of airborne biological density over each cave (Fig. 2b). We define emergence time at each cave as the maximum increase in the index of total airborne biological density (dZ/dt) over the cave domain in the ten hours surrounding sunset. Biologically this should correspond to time of the peak emergence when the greatest exodus occurs. If a cave produced multiple emergences, then we defined emergence time as the maximum increase of the first emergence. We visually inspected radar images to ensure these maxima were indeed associated with emergences of bats as opposed to weather, clutter, or other signals. Nights in which emergences were obscured by weather were excluded from the analysis. We converted time of emergence to offsets in minutes from local sunset to normalize times across caves and dates.

We observed emergence of Brazilian free-tailed bats from Frio Cave on 10 nights from 22 June–1 July in 2011 to confirm radar observations. Visual estimates of timing of emergence were similar to those derived from radar. Radar reflectivity factor values can be assumed to derive primarily from Brazilian free-tailed bats given that other bat species that may use these caves occur in much lower densities, fly at lower altitudes, and do not emerge in dense columns.

### Seasonal Climate and Emergence Behavior

To test our hypothesis that timing of emergence depends on summer climatic conditions, we averaged daily emergence time offsets across the 30-day study period for each site and averaged across sites to get a regional average of emergence time in each study year (2001–2011) (Table 1). We represented summer climatic conditions using the Palmer Drought Severity Index (PDSI) to measure combined effects of precipitation and temperature. PDSI is a measure of long-term drought and weekly reports are weighted by conditions in preceding weeks [32]. PDSI values range from −4.5 (extreme drought) to +4.5 (extreme moist). We averaged weekly indices of PDSI reported in climate divisions (Texas divisions 6,7,9) for sites during the 30-day study period from 2001–2011 (available online from NOAA's drought monitoring program (http://www.cpc.ncep.noaa.gov/products/monitoring_and_data/drought.shtml) (Table 2). We averaged across divisions for sites bordering multiple divisions. We used least-squares linear regression with the regional average of timing of emergence offset as the response variable and regional average of PDSI as the explanatory variable to determine the relationship between summer climatic condition and emergence behavior of bats. No evidence of temporal autocorrelation among years was evident based on visual inspection of residuals using the auto-correlation function (acf) in Program R [33]

**Table 1:** Time of emergence offset (minutes from sunset) of Brazilian free-tailed bats (*Tadarida brasiliensis*) at five maternity colonies in south-central Texas averaged from June 15–July 15.

	Bracken	Davis	Frio	Ney	Rucker	Grand Average
**2001**	9.6±23.8	33.8±18.1	45.5±11.8	45.1±13.0	49.40±21.5	36.7
	(27)	(28)	(27)	(25)	(18)	
**2002**	−38.3±53.9	−10.2±43.8	−26.4±46.5	−29.8±48.5	–	−26.2
	(19)	(16)	(20)	(21)	–	
**2003**	−46.9±46.6	−20.4±35.5	6.9±38.7	8.4±30.1	–	−13.0
	(22)	(20)	(19)	(19)	–	
**2004**	−62.6±48.7	−50.5±31.6	−2.6±45.0	−0.9±43.4	–	−29.2
	(30)	(27)	(26)	(29)	–	
**2005**	−77.0±26.2	−30.2±26.3	−78.0±37.4	−28.8±24.0	−65.6±22.1	−55.9
	(30)	(28)	(30)	(28)	(30)	
**2006**	−143.4±31.5	−86.2±24.2	−99.9±26.1	−82.9±54.7	−90.3±48.3	−100.6
	(29)	(28)	(28)	(26)	(28)	
**2007**	22.3±18.3	42.3±13.7	37.9±14.0	47.4±16.6	37.2±15.9	37.4
	(29)	(30)	(30)	(29)	(29)	
**2008**	−102.4±53.1	−104.4±29.4	−84.7±52.5	−58.1±42.3	−97.2±31.6	−89.4
	(29)	(27)	(23)	(27)	(17)	
**2009**	−101.2±29.2	−70.1±43.9	−59.6±43.8	−44.3±27.1	−64.4±28.4	−67.9
	(30)	(30)	(30)	(30)	(30)	
**2010**	26.9±36.0	26.6±44.5	42.0±29.6	80.3±23.3	38.1±34.1	43.4
	(29)	(28)	(30)	(28)	(28)	
**2011**	−73.8±35.3	−110.3±40.1	−20.2±16.3	−45.1±32.3	45.4±19.5	−58.9
	(30)	(30)	(28)	(30)	(28)	

Means shown with ± standard deviation. Samples sizes of days given below in parentheses. Missing values were due to days obscured by weather or missing radar data for a given date.

No emergences were visible on radar at Rucker cave during 2002–2004.

**Table 2:** Palmer Drought Severity Index scores associated with five maternity colonies of Brazilian free-tailed bats (*Tadarida brasiliensis*) in south-central Texas averaged from June 15-July 15.

	Bracken	Davis	Frio	Ney	Rucker	Grand Average	Year Type
**2001**	0.00	0.00	−0.42	0.00	0.00	−0.08	Normal
**2002**	−0.94	−0.94	−0.83	−0.94	–	−0.91	Normal
**2003**	0.00	0.00	1.30	0.00	–	0.33	Normal
**2004**	1.00	1.00	2.17	1.00	–	1.29	Wet
**2005**	0.00	0.00	0.00	0.00	0.00	0.00	Normal
**2006**	−3.75	−3.75	−3.94	−3.67	−3.75	−3.74	Dry
**2007**	2.38	2.38	3.00	2.38	3.25	2.68	Wet
**2008**	−2.50	−2.50	−2.75	−2.50	−2.50	−2.55	Dry
**2009**	−4.25	−4.25	−4.33	−4.25	−4.00	−4.22	Dry
**2010**	1.25	1.25	2.20	1.25	1.00	1.39	Wet
**2011**	−4.5	−4.5	−4.5	−4.5	−4.5	−4.5	Dry

Year type designates grouping labels assigned for analysis of covariance models.

### Daily Temperature and Emergence Behavior

We compared five a priori linear regression models using generalized least squares to determine how daily weather conditions influenced timing of emergence given yearly drought conditions. For this analysis, we calculated daily averages of emergence time offsets by averaging values for each of the five maternity colonies on each of the 30 days in 2001–2011 for the response variable. The five a priori models included a null model (emergence time constant), a main effects model with daily surface temperature as a predictor, a main effects model with a categorical variable of years classified as dry (PDSI score<−1), normal (PDSI score = −1 to 1), or wet (PDSI score>1), and parallel and varying slopes models with daily temperature as a continuous predictor and type of year (dry, normal, wet) as categorical predictor (Table 3). We used daily surface temperatures available from NOAA meteorological stations in the region from five stations situated near each colony from 2005 to 2011. Meteorological data were only available from two stations (KSAT and KHDO, near Bracken and Ney caves, respectively) from 2001–2004. Averaged values of daily temperatures from KSAT and KHDO were highly correlated (r = 0.94) with values from the other three stations in years in which we had data from all five stations (2005–2011). Therefore, we used data from KSAT and KHDO to represent daily temperature values in the region from 2001–2004.

**Table 3:** Model selection results from 5 a priori models of how timing of emergence by Brazilian free-tailed bats (*Tadarida brasiliensis*) responds to variation in daily surface temperature in south-central Texas in different summer climate conditions.

Model	ΔAIC	AIC weights	Para-meters
EmergeTime∼temperatureC* year_type	0	0.98	6
EmergeTime∼temperatureC+year_type	7.6	0.02	4
EmergeTime∼year_type	36.2	0.00	3
EmergeTime∼temperatureC	47.3	0.00	2
EmergeTime∼1 (null)	70.2	0.00	1

Visual inspection of residuals using the auto-correlation function (acf) in Program R [33] suggested significant temporal autocorrelation in residuals of models with standard correlation structure. Following suggestions by Zuur et al. [34], we used the varying slopes model and fit five models with increasing complexity on correlation structure, including no auto-correlation structure, compound symmetry auto-correlation structure, auto-regression of order 1 structure, and two forms of moving average auto-correlation structure. Results from AIC model comparisons demonstrated strong support for much better fit with the model of auto-regression of order 1 structure (99% AIC weights). Therefore, we present results comparing our five a priori biological models described above using an auto-regressive model of order 1 as the alternative correlation structure to account for temporal autocorrelation [34]. We tested for effects of moonlight by comparing emergence timing on nights with full and new moon and found no evidence to support a lunar effect (t = 0.06, df  =  19.13, p  = 0.95).

Analyses were conducted in R.12. [33] with packages nlme [35] and ggplot2 [36].

## Results

### Seasonal Climate and Emergence Behavior

**Figure 3:**
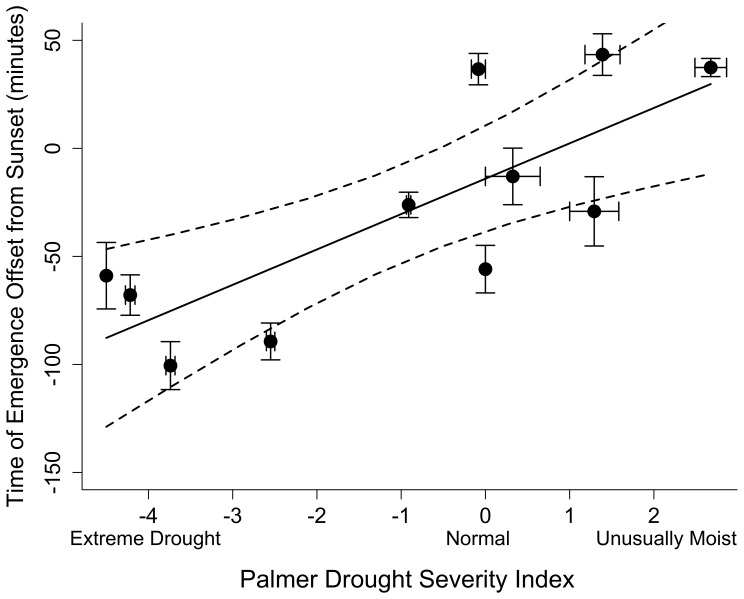
Climate and Emergence Behavior. Mean time of emergence (offset in minutes from local sunset) of Brazilian free-tailed bats (*Tadarida brasiliensis*) from five maternity colonies in south-central Texas was significantly earlier in the evening during years with extreme drought (R^2^ = 0.62, df = 9). Data were averaged across 30 days from June 15–July 15 during peak lactation from 2001–2011 from 5 maternity colonies. Error bars show standard errors around means for both drought severity (x-bars) and emergence time offset (y-bars) for each year.

Brazilian free-tailed bats emerged to forage significantly earlier in the evening during drought events than in years with normal to unusually moist conditions (p<0.01) (Fig. 3). The estimated slope coefficient equaled 16.38 minutes (95%CL: 8.0, 24.7), indicating that bats emerged roughly 16 minutes later in the evening with each unit increase (i.e. increasing moistness) in PDSI. During extreme drought events (PDSI = −4.5) bats emerged as early as 88 minutes before sunset (95%CL: −129, −47), whereas in unusually wet years (PDSI = 2.65), bats emerged as late as 30 minutes after sunset (95%CL: −11, 71).

### Daily Temperature and Emergence Behavior

**Figure 4:**
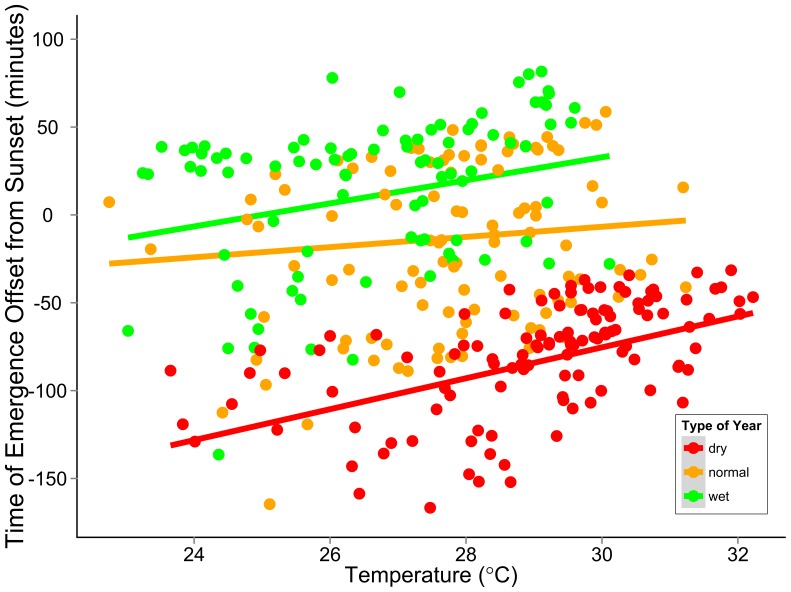
Daily Temperature and Emergence Behavior. Relationship of timing of emergence (offset in minutes from local sunset) of maternity colonies of Brazilian free tailed bats (*Tadarida brasiliensis*) and daily surface temperature (°Celsius). Predicted lines correspond to the best-fit varying slopes model with different intercepts for dry, normal and wet years and different slopes for the relationship of emergence time offset and daily temperature.

The varying slopes model after accounting for significant temporal auto-correlation in model residuals was the best fit according to AIC (AIC weight = 0.98) and indicates that timing of emergence was significantly different in dry, normal, and wet summers and that the relationship between daily timing of emergence and temperature depends on summer climate type (Fig. 4; Table 3). The estimate of correlation of residuals separated by one day was 0.86 (95% CL: 0.79,0.90). The relationship between onset of emergence and daily surface temperature was steepest during dry years, when bats emerged 9 minutes (95% CL: 5,13) later for every 1°C increase in daily surface temperature (Fig. 4). The relationship between onset of emergence and daily surface temperature was similar in wet years, when bats emerged 7 minutes (95% CL: 3, 10) later for every 1°C increase in daily surface temperature (Fig. 4). There was no significant relationship between onset of emergence and daily temperature during years of normal summer climate conditions (slope coefficient = 3; 95% CL: −1,7, p = 0.17) (Fig. 4).

## Discussion

Our results demonstrate a strong association between climatic conditions and emergence behavior in Brazilian free-tailed bats. Bats emerged earlier in years that were characterized by severe drought conditions and later in years with moist conditions (Fig. 3). This pattern matches our predictions and supports the hypothesis that timing of emergence in bats is an adaptive tradeoff between meeting foraging needs and decreasing risks of predation and competition [13]. Drought conditions are associated with lower insect availability [20] and have been linked to lower reproductive success [6] and lower annual survival [37] in some bat species. Our results suggest that bat colonies respond to variation in extrinsic conditions that affect physiological stress by emerging to forage earlier, sometimes well before sunset.

Daily weather also influenced timing of emergence such that bats emerged later on hotter days in both dry and moist years (Fig. 4). Foraging success may be highest on hot days because of the underlying relationship with nocturnal insect activity and temperature [21]. This relationship was consistent in both drought and moist years, suggesting that bats responded similarly in both types of climatic extremes. Surprisingly, there was no relationship with daily temperature and onset of emergence in years with normal moisture levels (Fig. 4). In general, there was much more variance in timing of emergence during normal years. The results of our analysis of daily patterns of emergence correspond well with the results of our analysis on summer climatic conditions and emphasize the importance of the role of both longer-term seasonal climate and short-term daily weather on animal behavior.

Phenotypic plasticity in response to climate can be an adaptive response that mediates impacts of changing climate on wild populations [38]. Emerging earlier is likely a signal of stressful conditions for bats, which has the potential to reduce individual fitness. Alternatively, early emergence could be a compensatory behavior such that bats respond to poor conditions by increasing foraging times without suffering loss of fitness. Understanding how variation in time of emergence relates to individual survival and reproduction or population declines is necessary to predict how climatic conditions will influence bat populations over the long term. Reproductive success and survival have been shown to vary with climatic conditions in other bat species [6], [37], but how plasticity in emergence behavior affects fitness and ultimately population growth is unknown.

One way to determine if emergence behavior in response to climate conditions results in changes in population growth would be to estimate population sizes of bat colonies over the same time frame in order to test whether years following severe drought were associated with significant population declines (i.e. N_t+1_≪N_t_). We are currently working on estimating aerial densities of bats directly from radar products [24]. The strength of the radar signal is related to the density of animals in the radar sampling volume and can be used to estimate animal densities, given certain assumptions [24]. Using radar reflectivity to estimate population sizes at these colonies will allow us to test how phenotypic response to climate influences population dynamics and will provide a useful means for long-term monitoring of bat population trends.

Past studies have investigated the functional significance of timing of emergence by assessing adaptive trade-offs, comparing foraging habits, and determining differences in age and reproductive conditions [13], [14]. Because we estimated timing of emergence from radar signals, our measure of onset of emergence is not directly comparable to other reported measures, such as visual assessment of first appearance or median emergence time [13], [39]. If anything, our measures of emergence timing may be biased late because there will always be a time lag between when bats leave the cave and when they are flying high enough to be detected by radar. Our radar-derived measure of maximum dZ/dt would be most similar to median emergence time, which is recognized as a better metric for measuring emergence behavior, than time of first appearance [39].

Brazilian free-tailed bats in our study emerged substantially earlier than reported emergence times of other bats. In a review comparing emergence times of bats, Jones and Rydell [13] provide timing of first appearance and median emergence for 66 species of bat from 11 families. In only four species, did time of emergence occur before local sunset and the earliest reported emergence was only 16 minutes before sunset [13]. Emergence was earliest in species like Brazilian free-tailed bats that have high flight speeds and depend on aerial insects [13]. Our results show that in moist years Brazilian free-tailed bats emerged 30 minutes after sunset and in dry years bats emerged as early as 1.5 hours before sunset (Fig. 3).

Our results were similar to emergence times reported for Brazilian free-tailed bats from Frio Cave in 1996 and 1997 [18] and from Davis, Frio and Ney caves in 2007 [17], supporting the efficacy of radar-based methods to measure emergence behavior in this species. Reichard et al. [17] reported emergence times for captured individuals in different reproductive classes during early summer. Median emergence time for lactating bats, which were the majority of captured bats (65%) in that season, was 47 minutes after sunset [17]. Average of median emergence times for Davis, Frio and Ney caves in our dataset in 2007, which likely roughly corresponds to the ‘early summer’ period used in the Reichard et al. [17] study, was quite similar at 41 minutes (means shown in Table 1). It may seem surprising that our data, which requires that the emerging column of bats has gained sufficient altitude to be detected, reports an earlier median time than data from individual bats captured at the cave entrance. The Reichard et al. [17] study had low samples size of nights as emergence was measured only twice monthly because of logistical challenges of being physically present and concerns about disturbing bats while capturing at entrances [17]. In contrast, we were able to measure emergence times for most days for the 30-day period of interest without any disturbance (Table 1). Our analysis on daily variation on emergence behavior shows that there is considerable daily variation in timing of emergence (Fig. 4), which could explain reported differences between the two studies due to sample sizes. Our results confirm suggestions by both Reichard et al. [17] and Lee and McCracken [18] that timing of emergence in Brazilian free-tailed bats is influenced by environmental cues, such as climate and weather conditions.

Our study is the first to use a sufficiently long yearly time series to assess how annual variation in climate conditions influences emergence behavior in bats. Annual variation in emergence times demonstrates that plasticity in emergence behavior of bats is a response to environmental cues by which bats can alter foraging strategies to meet energy needs. Our data suggest that bats respond to both daily and seasonal conditions and that drought conditions are associated with riskier behaviors of emerging earlier. Emergence timing may be a useful long-term indicator of response to climate change by bats, particularly in arid environments. Future studies should aim to link the fitness consequences of emergence behavior response to climate and weather patterns.

We used remote-sensing technology and freely available climatic indices to associate animal behavior with annual variation in climate and daily weather conditions. Numerous studies have investigated timing of emergence in bats, as it is an easily measured behavioral signal [13]. However, without a remote-sensing capability to measure timing of emergence, the ability to assess how daily weather or seasonal climatic conditions influence group behavior had yet not been attempted. By using the archived NEXRAD radar network to measure emergence timing, we were able to monitor animal behavior at a temporal and spatial scale concordant with determining how animal aggregations respond to annual and daily variation in weather conditions. In our analysis, we used 11 years of data because the entire 20-year NEXRAD archive has not yet been processed in a user-friendly format for biological research. Access to the entire NEXRAD archive in a mosaicked and composite format would facilitate future ecological research and support monitoring of animal response to weather and climate.
